# Chinese Breast Milk Fat Composition and Its Associated Dietary Factors: A Pilot Study on Lactating Mothers in Beijing

**DOI:** 10.3389/fnut.2021.606950

**Published:** 2021-05-28

**Authors:** Wei Wu, Adi Balter, Victoria Vodsky, Yatreb Odetallh, Gai Ben-Dror, Yumei Zhang, Ai Zhao

**Affiliations:** ^1^Department of Nutrition and Food Hygiene, School of Public Health, Peking University, Beijing, China; ^2^Infant Nutrition R&D, IFF Health, Migdal HaEmeq, Israel; ^3^Infant Nutrition, Enzymotec Ltd., Migdal HaEmeq, Israel; ^4^Vanke School of Public Health, Tsinghua University, Beijing, China

**Keywords:** breast milk, triacylglycerols, maternal diet, fatty acids, OPL

## Abstract

Regional differences were found in breast milk composition. This study intended to profile the composition of fatty acid (FA) and triacylglycerol (TAG) in Chinese breast milk and to explore its association with maternal diet. Breast milk samples and data of 52 lactating women at 60–90 days postpartum were collected. The FA composition was measured using gas chromatography–flame ionization detection (GC-FID), and the TAG profile was detected by an ultra-performance liquid chromatography system, coupled with accurate-mass quadrupole time-of-flight mass spectrometer. A semi-quantitative food intake frequency questionnaire and a one-time 24-h dietary recall were used to evaluate the previous month's and the short-term dietary intake, including dietary patterns, food groups, and nutrients. Oleic–palmitic–linoleic (OPL) is the most predominant TAG within the Chinese human milk, followed by oleic–palmitic–oleic (OPO), with an average OPL-to-OPO ratio of 1.35. Linoleic acid (LA) and oleic acid (OA) accounted for 23.9 and 32.0% of the total FAs, respectively. Among the food groups consumed during the preceding month, LA content was positively associated with the consumption of soybeans and soybean products (*r* = 0.311, *p* = 0.030), whereas a negative correlation was identified with seafood consumption (*r* = −0.302, *p* = 0.030). Negative correlations were found between the OA content and the consumption of soybeans and soybean products (*r* = −0.363, *p* = 0.009), livestock and poultry meat (*r* = −0.375, *p* = 0.006), nuts (*r* = −0.305, *p* = 0.028), as well as cooking oil (*r* = −0.445, *p* = 0.001). No significant associations were identified between the LA and OA contents and the dietary patterns. This study confirmed a high OPL level in Chinese breast milk and revealed associations of FAs with maternal dietary intake.

## Introduction

Human milk is considered to be the best source of nutrition for infants, providing adequate amounts of highly bioavailable nutrients and many bioactives to support the growth and development of infants (1, 2). Breast milk is an undeniably unique natural source of nutrition for infants. The composition of breast milk changes during lactation, during each feed, and over 24 h and differs between individuals (3–5). Breast milk lipids are present in human milk fat globules, which constitute 3–7% of the total fat content (6). About 98 g/100 g of the total lipids in human milk are triacylglycerol (TAG), and of these, 90 g/100 g are fatty acids (FAs) (6). A great deal of literature has demonstrated that breast milk fats are some of the most mutable nutrients in breast milk (7, 8).

Based on our previous study and other comparative studies, the lipid composition of Chinese breast milk is substantially different from breast milk in Western countries (9, 10). Chinese breast milk is characterized by a high level of *n*-6, but a relatively low level of *n*-3 FAs (10). TAG, the major source of energy in human milk, comprised three fatty acids, selectively esterified to the glycerol skeleton at three stereospecifically numbered (sn) positions. Several recent papers have revealed that the structure of TAG in Chinese breast milk is also different from other countries, which may result in different physiological and metabolic implications in breastfed infants. In countries such as Denmark and Spain, the predominant structure of TAG has been reported to be oleic–palmitic–oleic (OPO); however, oleic–palmitic–linoleic (OPL) is the predominant structure observed in Chinese breast milk (11–13).

The genetic background, physical status, and the dietary intake of lactating women, as well as the different characteristics of their infants, might all contribute to the variations in human breast milk (14–17). Although several studies conducted in China have explored the reasons for the heterogeneity in breast milk fat composition, maternal dietary factors need further confirmation due to the small sample size, heterogeneous nature of the samples (samples collected from a wide time span across the lactation period and mothers with different socio-demographic characteristics), non-standard collection procedures, and the inappropriate storage of breast milk in these studies (11, 18, 19). Particularly, China is known for its unique postpartum dietary custom, called “Zuo yuezi” in Chinese (20). Based on Chinese traditional cultural beliefs and theories (21), this cultural puerperal practice encourages the consumption of “hot” foods, such as chicken soup, pig's trotter soup, and brown sugar, within the first month postpartum while limiting the consumption of “cold” meals, for example seafood and fresh fruits. Different dietary habits have been shown for Western countries as well. For example, a cohort study conducted in the USA that followed pregnant women for 1 year after delivery suggested that breastfeeding mothers were more likely to switch to the “prudent” dietary pattern, featuring at both 3 and 12 months postpartum a high consumption of fruits and vegetables, whole grains, beans, nuts, fish and chicken (not fried), water, and low-fat dairy, than were non-breastfeeding mothers (22). Dos et al. found that Brazilian lactating mothers tend to change their dietary habits to a higher consumption of rice in comparison to their pregnancy period (23). Compared with the Western world, the Chinese postpartum dietary custom might provide clues to the variations in the composition of Chinese breast milk fat.

In this study, mature breast milk samples and data of dietary intake were collected from healthy Chinese lactating women at 60–90 days postpartum. Breast milk fats were analyzed to determine the composition of FAs and TAGs and explore its association with maternal diet.

## Materials and Methods

### Participants

This work is part of The Dietary Effects on Breast Milk Composition and Its Association with Infants' Health cohort study, conducted in Beijing. In this cohort, newborns and their lactating mothers were recruited after birth in maternal and child hospitals from May 2018 to July 2018 and followed for 1 year postpartum. The inclusion criteria of healthy mothers, based on volunteer participation, were: (1) age of 18–45 years; (2) full-term delivery (over 37 weeks); (3) single birth with a healthy baby; (4) without nipple or lacteal gland diseases; (5) non-smoker and non-drinker; and (6) intention to follow exclusive breastfeeding for 6 months. Women with metabolic disease, use of hormones in the last 3 months, and experience of postpartum depression or other mental illnesses were excluded. Finally, breast milk samples and data collected from 52 lactating women at 60–90 days postpartum were used for the analysis. All procedures of this study were approved by the Medical Ethics Research Board of Peking University based in Beijing, China (no. IRB00001052-16038).

### Breast Milk Collection and Storage

All participating mothers were advised to follow their normal dietary habits before breast milk collection. Breast milk sampling was standardized for all subjects as follows in order to avoid the influence of circadian and within-feed variations on breast milk components. On the day of investigation, the women were instructed to feed their babies and empty their breast between 6 and 7 a.m. Sample collection was fixed at the second feeding in the morning (9:00–11:00 a.m.). After cleaning with warm water, one single breast was emptied by hand, by a trained investigator. Foremilk, mid-milk, and hindmilk were gently mixed to represent one complete feed. Then, a total of 30 ml was secured, separated into three conical 15-ml polypropylene tubes, and immediately kept at −20°C at the local maternal and child hospital. Within 24 h from collection, the samples were transferred to −80°C by cold chain transportation (at −20°C).

### Data Collection

An interviewer-administered questionnaire was used to collect socio-demographic characteristics, lifestyle and behavioral information, the history of pregnancy and delivery, as well as dietary intake. Metabolic equivalents (METs) were calculated from the International Physical Activity Questionnaire (short version) to assess the intensity of physical activities.

Short-term dietary intake was based on a one-time 24-h dietary recall. This specific dietary recall was chosen as the dietary habits of lactating women during the confinement period are considered to have less day-to-day variations on a short-term basis (24). During the interview, trained interviewers asked the participants to report all food and beverages, including seasonings and supplements, consumed the day before the interview. Quantity of the item consumed, time, and a description of the meal were recorded. Intakes of total energy and fat were calculated based on the Chinese Food Composition Table 2009 and the nutritional information on the food packaging (25).

Dietary intakes over the previous month were assessed by a 21-item semi-food frequency questionnaire (FFQ). According to the Balanced Diet Pagoda for Lactating Women (26), the food items were categorized into the following groups: (1) cereals, (2) tubers, (3) fresh vegetables, (4) fresh fruits, (5) livestock and poultry meat, (6) seafood, (7) freshwater food, (8) eggs, (9) dairy products, (10) soybeans and soybean products, (11) mixed beans, (12) nuts, and (13) cooking oil. The average daily intake (in grams/day) of each food category was estimated by the average daily intake frequency multiplied by the average intake amount. Measurement aids including standard bowls, plates, and spoons, as well as a picture booklet of the common foods consumed in China, were used to standardize the quantification of food items.

Maternal height and weight were measured in field by trained health professionals during visits at maternal and child hospitals. Body mass index (BMI) was calculated as weight/height^2^ (in kilograms/square meter). The postpartum weight retention (in kilograms) was calculated as the current weight minus the self-reported pre-pregnancy weight. The gestational weight gain (in kilograms) was calculated as the self-reported pre-delivery weight (2 weeks before delivery) minus the self-reported pre-pregnancy weight.

### Lipid Analysis

#### Materials and Reagents

TAG standards were purchased from Larodan Fine Chemicals AB (Sweden). Methyl ester standards were purchased from Nu-Chek (USA), and methyl tricosanoate (Sigma-Aldrich) was used as the internal standard. Methanol, acetonitrile, and isopropanol were all LC/MS grade. Chloroform and hexane were of high-performance liquid chromatography (HPLC) purity. All were purchased from Fisher Scientific (Fair Lawn, NJ, USA). Boron trichloride methanol 12% solution was purchased from Supleco, and sodium sulfate anhydrous and sodium hydroxide were purchased from Sigma.

#### Sample Preparation

Human milk total lipids were extracted according to the method described by Folch et al. (27) and Sündermann et al. (28). Briefly, 10 g of each human milk sample were dissolved in chloroform/methanol (2:1, *v*/*v*) mixture, shaken for 30 min at maximal speed, and centrifuged for 10 min at 7,000 rpm. The extract (organic phase) was equilibrated with one-fourth volume of the KCl solution (8.8 g/L), mixed vigorously, and centrifuged for 2 min at 5,000 rpm. The lower chloroform layer was filtered (0.22 μm PTFE filter) and evaporated, and the obtained total lipids were stored at −80°C for further analysis.

#### Fatty Acid Composition

FA methyl esters of breast milk total lipids were prepared by transferring 350 μl of the total lipid solution (100 mg/ml in chloroform/methanol, 95:5, *v*/*v*) into a sealable tube and evaporated at 60°C under nitrogen to constant weight. One milliliter of methyl tricosanoate 2 ml/ml in toluene (used as the internal standard) and 0.5 ml of NaOH in methanol (0.5 mol/L) were added and the mixture was heated for 7 min. Once cooled, 1 ml of boron trichloride solution was added and the mixture was covered with nitrogen, vortexed, and heated for 20 min. Following mixing with 2 ml of hexane and 1 ml of purified water, the upper layer was collected, dried over Na_2_SO_4_, filtered through a 0.2-μm filter, and was analyzed by GC (Agilent 7693) equipped with an autosampler, a flame ionization detector, and DB-WAX column (10 m × 0.1 mm × 0.1 μm; Agilent, USA). The running time was set to 17.3 min for each sample. The temperature of the injector and the detector was set at 240°C. The analysis of milk fat FAs was performed using a temperature gradient program from an initial temperature of 40°C, first raised by 25°C/min to 195°C, then raised by 3°C/min to 205°C, and then raised by 8°C/min to a final temperature of 230°C and kept for 5 min. Helium was used as a carrier gas with a flow rate of 0.3 ml/min, split ratio of 1:200, detector gas 40 ml/min hydrogen, and 400 ml/min air and 25 ml/min nitrogen. The identification of milk FAs was conducted by comparing the retention times of the GC peaks with corresponding known standards.

#### Triacylglycerol Composition

Twenty-five milligrams of breast milk total lipids of each participating mother was dissolved in 25 ml of chloroform/methanol mixture (2:1, *v*/*v*). The internal standard was added to the sample solution at a final concentration of 0.1 mg/ml, and the solution was then diluted with isopropanol to a final volume of 50 ml (final concentration of 0.5 mg/ml). The final solution with internal standard was subjected to analysis. The exact content of TAGs in each participant's total lipid fraction was determined based on the calibration curves of the TAG standard solutions prepared in the chloroform/methanol mixture and isopropanol solution of the internal standard.

The analysis of breast milk TAGs was carried out by an ultra-performance liquid chromatography (UPLC) system (Agilent 1290 infinity) equipped with an ACQUITY UPLC HSS T3 column (1.8 μm × 2.1 mm × 100 mm). The flow rate was 300 μl/min, the column temperature was set at 40°C and the sample chamber temperature at 20°C, and the injection volume was 1 μl for each analysis with a concentration of 0.5 mg/ml. The separation of milk TAGs was performed using acetonitrile as mobile phase A, whereas isopropanol was used as mobile phase B. Effective separation of milk TAGs was achieved with a binary gradient started with a decrease of phase A to 60% for 2 min and maintained for 2 min, then decreased to 50% for 11 min and held for 2 min, and then returned to the initial 100% for 1 min and equilibrated for 2 min. Ammonium formate Aq., (10%, 50 mM) was added after column separation for ionization in quadruple time-of-flight (Q-TOF). After the analysis of each sample, the column was flushed with the same binary gradient before the beginning of the next analysis.

A Q-TOF MS instrument (Agilent 6540 UHD Accurate-Mass, 6540) with an electrospray ionization (ESI) probe was used for the identification and quantification of the breast milk TAGs. Positive ion mode was used at an optimized condition as follows: capillary voltage, 3 kV; cone voltage, 0 V; drying gas, 8 L/min (nitrogen, 300°C); sheath gas, 8 L/min (nitrogen, 350°C); nebulizer, 35 psi; and fragmentor, 140 V. The mass was detected in the range of 50–1,700 *m*/*z* for a 0.5-s scan duration. The calibration curves were made with the TAG standards in the range of 0.5–100 μg/ml. The corresponding adduction peaks from the different milk TAG classes were detected under the positive ion full-scan ESI-MS analysis and the specific TAG species based on the ESI-MS/MS analysis. Using the MS mode, precise calculation of the molecular weight distribution of the TAGs, through the accurate mass of the quasi-molecular ion [M+NH_4_]^+^, was obtained. The relative concentration was calculated through dividing the peak area of an individual TAG by the sum of all the peak areas within the sample. The MassHunter Quantitative Analysis version B.07.01/Build 7.1.524.0 software (Agilent) was used for instrument control and analysis of the obtained data.

### Statistical Analysis

SAS version 9.3 (SAS Institute, Inc., Cary, NC, USA) was used for statistical analysis. Values are presented as the mean ± SD or median (25th and 75th percentiles) for skewed distribution data or percentages.

Factor analysis with a principal component method was used to explore the dietary pattern based on data of the average daily food intake *via* the semi-FFQ. We rotated the factors using an orthogonal transformation with a varimax option to achieve factors with greater interpretability. A combined evaluation of the eigenvalue (>1), scree plot, and interpretability of factors determined the number of components. The food items with a factor loading >0.60 were considered as the characteristic food of each dietary pattern. Factor scores were calculated for each individual in each dietary pattern by summing the intakes of the 15 food groups weighted by the factor loadings.

Pearson's correlation test was used to determine the association between breast milk FA composition and the maternal dietary intake. A *p*-value <0.05 was considered statistically significantly different.

## Results

### Basic Characteristics of 52 Pairs of Mothers and Infants

A total of 52 exclusively breastfeeding mothers participated in this study. The socio-demographic characteristics, lifestyle, and health-related indicators of the 52 pairs of mothers and infants are shown in Table 1. The lactating women were characterized by Han nationality (94.2%) and a high education level. The majority of the women experienced their first parity, and spontaneous delivery, demonstrating postpartum weight retention at the time of measurement.

**Table 1:** Basic characteristics of the 52 pairs of mother and infant.

**Characteristics**	**Description**	
**Mother**		
Age (years)		31.5 ± 4.7
Education level	Senior high school or below	12 (23.1)
	Bachelor's degree	23 (44.2)
	Master's degree or above	17 (32.7)
Household monthly income (RMB: yuan)	<3,000	13 (25.0)
	3,000–8,000	22 (42.3)
	>8,000	17 (32.7)
Parity	First	33 (63.5)
	Second	19 (36.5)
Physical activity level (METs)		420.5 (70.0, 1,290.0)
Current BMI		23.3 ± 3.5
Gestational weight gain (kg)		14.0 ± 5.9
Postpartum weight retention (kg)	<3	24 (46.3)
	3–5	9 (17.5)
	>5	19 (36.5)
**Infant**		
Gender	Male	29 (55.8)
	Female	23 (44.2)
Mode of delivery	Spontaneous labor	31 (59.6)
	Cesarean section	21 (40.4)
Birth weight (g)		3,440 (3,075, 3,630)

*Continuous variables were presented as the mean ± standard deviation or median (P25, P75) according to normality of data. Categorical variables were presented as N (%)*.

*MET, metabolic equivalents; BMI, body mass index*.

### Dietary Intake of Lactating Mothers

The dietary intake of the participating mothers is presented in Table 2.

**Table 2:** Dietary intake of lactating mothers (*n* = 52).

	**Recommendations*^a^***	**Median (P25, P75)**	**Mean (95% CI)**
**Nutrients (during the preceding 24 h)**			
Total energy (kcal/day)	2,600	1,575.0 (1,085.16, 2,135.29)	1,607.0 (1,422.0–1,792.1)
Fat (g/day)		62.19 (41.50, 84.90)	66.9 (57.8–76.0)
Fat (% TE)	20–30	36.66 (31.70, 44.26)	37.7 (35.0–40.5)
Protein (g/day)	80	51.72 (41.49, 85.98)	63.9 (54.8–73.1)
Protein (% TE)		15.71 (13.03, 17.39)	79.9 (68.5–91.3)
Carbohydrate (g/day)		206.20 (117.86, 251.96)	195.8 (169.7–221.9)
Carbohydrate (% TE)	50–60	48.61 (43.96, 56.18)	48.3 (45.0–51.6)
**Food groups (during the preceding month)**			
Cereals (g/day)	250–300	300.0 (200.0, 330.0)	307.2 (270.0–344.5)
Tubers (g/day)	75–100	21.4 (10.7, 50.0)	52.1 (29.8–74.4)
Fresh vegetables (g/day)	300–500	300.0 (200.0, 600.0)	401.3 (327.2–475.5)
Fresh fruits (g/day)	200–400	200.0 (100.0, 375.0)	292.0 (200.3–383.8)
Livestock and poultry meat (g/day)	75–100	92.9 (50.0, 200.0)	149.8 (101.8–197.8)
Seafood (g/day)	75–100^b^	15.5 (0.0, 46.5)	19.3 (7.9–30.7)
Freshwater food (g/day)		8.9 (0.0, 42.4)	33.8 (18.9–48.8)
Eggs (g/day)	50	64.6 (50.0, 100.0)	77.5 (64.3–90.7)
Dairy (g/day)	300–500	175.0 (22.5, 250.0)	200.8 (139.1–262.4)
Soybeans and soybean products (g/day)	25	21.9 (2.1, 47.5)	35.2 (22.3–48.1)
Mixed beans (except soybeans) (g/day)		0.9 (0.0, 9.3)	9.0 (3.9–14.1)
Nuts (g/day)	10	14.6 (0.0, 31.4)	25.9 (15.0–36.8)
Cooking oil (g/day)	25	24.0 (19.0, 34.0)	24.5 (21.4–27.6)

*CI, confidence interval; % TE, percentage of total energy*.

a *According to the dietary reference intakes of macronutrients and total energy for lactating women in Dietary Guidelines for Chinese Residents 2016 and food intakes in Balanced Diet Pagoda for Lactating Women*.

b *Recommended total intake of seafood and freshwater food*.

Compared with the dietary reference intakes of macronutrients for lactating mothers in the 2016 Dietary Guidelines for Chinese Residents (20), the carbohydrate intake of 55.8% of the participating mothers was below the lower limit, with 9.6% above the upper limit. Additionally, 67.3% of the mothers ingested less protein than the recommended 80 g per day. However, excessive fat intake occurred in 76.9% of the lactating mothers.

Comparison of the food consumption over the preceding month, with the recommendations of the Balanced Diet Pagoda for Lactating Women, revealed that 84.6% of the mothers consumed dairy products below the lower limit, while 65.4% consumed eggs excessively. Lactating women who had a livestock and poultry meat intake above the upper limit accounted for 38.5%, along with 28.9% for the total intake of seafood and freshwater food. The percentages of mothers who consumed nuts or cooking oil over the recommendations were 55.8 and 40.4%, respectively.

Four dietary patterns, which explained 57.2% of the whole variance of food intake, were extracted. Factor loading in these four dietary patterns can be seen in Supplementary Table 1. Dietary pattern 1 featured a high consumption of fresh fruits and dairy; dietary pattern 2 was characterized by a high intake of tubers and freshwater food; dietary pattern 3 was characterized by a high intake of nuts and mixed beans (except soybeans); and dietary pattern 4 featured a high consumption of livestock and poultry meat.

### TAG Profile of Mature Human Milk

**Figure 1:**
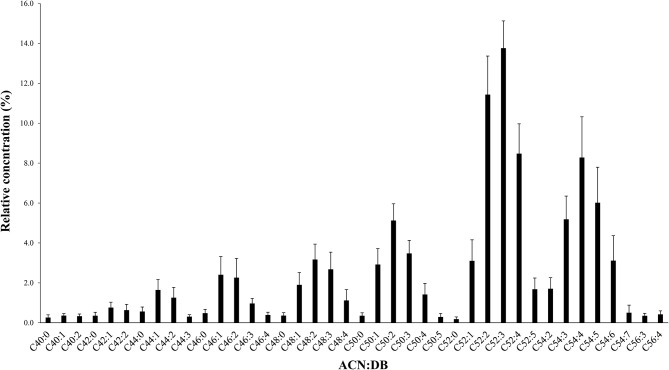
Molecular weight distribution of triacylglycerols (TAGs) with relative concentration (percent area) in breast milk. TAGs are presented as ACN:DBs (acyl carbon number:number of double bonds). Values are the relative concentration plus standard deviation of all breast milk TAGs. ACN:DB appears in the order of increasing molecular weight.

The average amount of total fat was found to be 5.67 ± 2.55 g per 100 g of breast milk sample. The identification and the quantitation of the TAG profiles of mature human milk from Beijing women were performed based on the acquisition data of Q-TOF-MS. Using the MS mode, precise calculation of the molecular weight distribution of the TAGs, through the accurate mass of the quasi-molecular ion [M+NH_4_]^+^, was obtained. The identified TAGs and their relative concentrations are presented in Figure 1. The relative concentration was calculated by dividing the peak area of an individual TAG by the sum of all the peak areas within the sample. Forty TAGs showing different ACN:DB (acyl carbon number:number of double bonds) were identified, with the lowest TAG level showing 0.18%. As shown in Figure 1, Chinese human milk contained higher levels of TAGs with long-chain and unsaturated fatty acids. Specifically, molecular species with ACN 52 constituted the largest ACN family (38.6%). The predominant ACN:DB was found to be C52:3 (13.8 ± 1.4%), followed by C52:2 (11.4 ± 1.9%).

The 17 most predominant TAG species were further identified and quantified based on MS/MS fragmentation and the calibration curves of external TAG standards (see Table 3). Using this methodology, different TAG species having similar ACN:DB could be accurately measured. For example, OPO and linoleic-palmitic-stearic (LPS) both share an ACN:DB of C52:2. However, OPO is far more abundant in human milk in comparison to LPS, showing 9.60 ± 2.31 vs. 1.29 ± 0.23 g/100g, respectively. This analysis further demonstrates that the most predominant TAG within the Chinese human milk is OPL, showing content of 12.28 ± 1.97 g/100 g and an OPL/OPO ratio of 1.35 (*p* < 0.001).

**Table 3:** Triacylglycerol species in mature human milk.

**TAG*^a^***	**ACN:DB**	**Formula**	**[M+NH_**4**_]^**+**^**	**Mean ± SD**	**Min**	**Max**
POLa	C46:1	C_49_H_92_O_6_	794.7233	2.54 ± 0.70	1.05	3.93
MOP	C48:1	C_51_H_96_O_6_	822.7546	1.17 ± 0.37	0.48	1.99
LaOO	C48:2	C_51_H_96_O_6_	820.7390	1.75 ± 0.56	0.78	3.68
PPO	C50:1	C_53_H_100_O_6_	850.7859	2.66 ± 0.76	1.30	4.89
OMO	C50:2	C_53_H_98_O_6_	848.7703	2.31 ± 0.50	1.44	4.02
PPL	C50:2	C_53_H_98_O_6_	848.7703	1.85 ± 0.39	1.02	3.12
MOL	C50:3	C_53_H_96_O_6_	846.7546	2.30 ± 0.54	1.46	4.37
OPS	C52:1	C_55_H_104_O_6_	878.8172	3.25 ± 1.08	0.60	6.78
OPO	C52:2	C_55_H_102_O_6_	876.8016	9.60 ± 2.31	3.43	15.64
LPS	C52:2	C_55_H_102_O_6_	876.8016	1.29 ± 0.23	0.59	1.78
OPL	C52:3	C_55_H_100_O_6_	874.7859	12.28 ± 1.97	7.55	17.00
LPL	C52:4	C_55_H_98_O_6_	872.7703	6.15 ± 1.64	3.06	10.68
OOO	C54:3	C_57_H_104_O_6_	902.8172	2.39 ± 0.72	0.53	3.91
LOO	C54:4	C_57_H_103_O_6_	900.8016	4.30 ± 1.71	1.64	8.82
LOL	C54:5	C_57_H_100_O_6_	898.7859	4.16 ± 1.37	1.78	7.74
OOLn	C54:5	C_57_H_100_O_6_	898.7859	2.34 ± 0.43	1.57	3.88
LLL	C54:6	C_57_H_98_O_6_	896.7703	9.03 ± 2.99	3.74	16.26

*TAG, triacylglycerol; SD, standard deviation; ACN, acyl carbon number; DB, number of double bonds; Bu, butyric acid; Ca, capric acid; La, lauric acid; M, myristic acid; P, palmitic acid; S, stearic acid; O, oleic acid; L, linoleic acid*.

a *TAGs do not represent positional distribution; for example, “OPO” stands for both OPO and OOP*.

### Fatty Acid Composition of Human Milk Fat

Table 4 presents the total FA composition of breast milk fat. Palmitic acid (PA, C16:0) accounted for over half of the total saturated fatty acids (SFAs). Monounsaturated fatty acids, dominantly composed of oleic acid (OA, C18:1n9), accounted for 36.5% of the total FAs detected, whereas polyunsaturated fatty acids (PUFAs) constituted 27.7%. The predominant ingredients of *n*-6 and *n*-3 PUFAs were linoleic acid (LA, C18:2n6) and alpha-linolenic acid (ALA, C18:3n3), respectively. The LA/ALA ratio is 16.3:1.

**Table 4:** Fatty acid composition in breast milk.

**Chemical expression**	**Designation**	**Mean ± SD**
C8:0	Caprylic	0.22 ± 0.04
C9:0	Non-anoatenoic	0.02 ± 0.01
C10:0	Capric	1.34 ± 0.31
C11:0	Undecylic	0.02 ± 0.01
C12:0	Lauric	4.34 ± 1.57
C12:1	Dodecanoatenoic	0.02 ± 0.01
C14:0	Myristic	3.81 ± 1.43
C14:1	Myristoleic	0.09 ± 0.05
C15:0	Pentadecanoatenoic	0.16 ± 0.07
C16:0	Palmitic	19.61 ± 1.97
C16:1	Palmitoleic	1.87 ± 0.42
C17:0	Margaric	0.24 ± 0.08
C18:0	Stearic	5.56 ± 1.00
C18:1n9	Oleic	32.04 ± 3.33
C18:1n11	Vaccenic	1.70 ± 0.34
C18:2n6	Linoleic	23.91 ± 4.19
C18:3n6	Gamma-linoleic	0.15 ± 0.05
C18:3n3	Alpha-linolenic	1.47 ± 0.94
C20:0	Arachidic	0.23 ± 0.08
C20:1n9	Eicosenoic	0.43 ± 0.10
C20:2n6	Eicosadienoic	0.40 ± 0.09
C20:3n6	Homo-g-linolenic	0.35 ± 0.07
C20:4n6	Arachidonic, ARA	0.55 ± 0.11
C20:3n3	Eicosatrienoic	0.05 ± 0.02
C20:5n3	Eicosapentaenoic, EPA	0.06 ± 0.03
C22:0	Behenic	0.13 ± 0.08
C22:1n9	Euricic	0.37 ± 0.16
C22:2n9	Docosadienoic	0.05 ± 0.02
C22:4n6	Docosatetraenoic	0.14 ± 0.12
C22:3n9	Docosatrienoatenoic	0.11 ± 0.06
C22:5n6	Docosapentaenoic	0.06 ± 0.04
C22:5n3	Docosapentaenoic	0.13 ± 0.05
C24:0	Lignoceric	0.11 ± 0.05
C22:6n3	Docosahexaenoic, DHA	0.33 ± 0.17
C24:1n9	Nervonic	0.05 ± 0.02
Total SFA		35.7 ± 3.69
Total MUFA		36.5 ± 3.68
Total PUFA		27.7 ± 4.39
Total *n*-3 PUFA		2.02 ± 0.99
Total *n*-6 PUFA		25.6 ± 4.23
Total *n*-9 PUFA		0.14 ± 0.08

*The profiles of the fatty acids in breast milk were presented with each fatty acid weight percentage of total fatty acids (% wt/wt)*.

*SD, standard deviation; SFA, saturated fatty acid; MUFA, monounsaturated fatty acid; PUFA, polyunsaturated fatty acid*.

### Associations of Dietary Intake With the Fatty Acid Content in Breast Milk

Table 5 presents the associations of the contents of LA and OA in breast milk with the maternal dietary intake of total energy and fat, consumption of different food groups, and dietary patterns.

**Table 5:** Associations of maternal dietary intake with breast milk contents of linoleic acid and oleic acid.

	**Linoleic acid**		**Oleic acid**	
	**Correlation coefficient**	***P*-value**	**Correlation coefficient**	***P*-value**
**Nutrients (during the preceding 24 h)**				
Total energy (kcal/day)	0.180	0.203	−0.055	0.699
Fat (% TE)	−0.052	0.713	0.134	0.343
**Food groups (during the preceding month)**				
Cereals (g/day)	−0.043	0.764	−0.165	0.242
Tubers (g/day)	0.021	0.882	−0.138	0.329
Fresh vegetables (g/day)	0.068	0.630	−0.093	0.235
Fresh fruits (g/day)	−0.009	0.949	−0.036	0.799
Livestock and poultry meat (g/day)	0.209	0.137	−0.375	0.006
Seafood (g/day)	−0.302	0.030	0.043	0.763
Freshwater food (g/day)	0.104	0.464	−0.217	0.123
Eggs (g/day)	0.032	0.822	−0.004	0.976
Dairy (g/day)	−0.181	0.200	−0.045	0.753
Soybeans and soybean products (g/day)	0.311	0.030	−0.363	0.009
Mixed beans (except soybeans) (g/day)	−0.222	0.114	0.163	0.247
Nuts (g/day)	0.112	0.429	−0.305	0.028
Cooking oil (g/day)	0.061	0.665	−0.445	0.001
**Dietary patterns (during the preceding month)**				
Pattern 1	0.090	0.524	−0.261	0.062
Pattern 2	0.032	0.821	−0.222	0.113
Pattern 3	−0.041	0.772	−0.012	0.935
Pattern 4	−0.153	0.280	0.011	0.936

*% TE, percentage of total energy*.

Associations of the contents of LA and OA with the intake of total energy and fat during the preceding 24 h did not meet the threshold for statistical significance.

Among the food groups consumed during the preceding month, the LA content was positively associated with the consumption of soybeans and soybean products, whereas a negative correlation was identified with seafood consumption. As for OA, negative correlations were observed between the OA content and consumption of soybeans and soybean products, livestock and poultry meat, nuts, as well as cooking oil. No significant associations were found between the contents of LA and OA and the four dietary patterns.

Associations between the contents of the other FAs and the dietary intake are displayed in the form of correlation coefficient heat maps (Supplementary Figures 1A–C). For example, the ALA content was positively associated with egg consumption, and the arachidonic acid (ARA, C20:4n6) content was positively associated with the consumption of freshwater food and fresh vegetables. A positive association of the PA content with dietary pattern 4, featuring a high consumption of livestock and poultry meat, was also found.

## Discussion

The current work aimed to explore the potential correlations between the fat composition of Chinese breast milk and the maternal dietary factors. This study confirmed that the predominant TAG species in Chinese breast milk were OPL, followed by OPO. We found that the LA content in breast milk was positively correlated with the consumption of soybeans and soybean products and negatively correlated with seafood consumption during the preceding month. We also observed negative correlations between the OA content and the consumption of soybeans and soybean products, livestock and poultry meat, nuts, as well as cooking oil.

### Characteristics of Chinese Breast Milk Fat Fraction

Exploring the TAG profiles of Chinese breast milk revealed that the most predominant TAG species is OPL, followed by OPO, leading to an OPL/OPO ratio above one. These results confirm similar findings from other studies for Chinese human milk (11, 18, 19, 29), but show an opposite trend in comparison to studies of breast milk from many Western countries, such as Finland, Spain and Italy (30–33), in which OPO was found to be the predominant TAG species with the OPL/OPO ratio below one.

Evaluation of the FA composition demonstrated that the proportion of *n*-6 PUFAs is within the range of the *n*-6 PUFA levels reported by other Chinese breast milk studies (10, 34), but is evidently higher than that previously observed in Western countries, including Italy, Germany, Finland, and Spain (10, 35, 36), as well as some Asian countries such as South Korea, Malaysia, and Japan (8, 36, 37).

The LA content (23.9%) and the ratio of LA to ALA (16.3:1) found in this study are both in agreement with data reported in China (34, 38), while higher than the values indicated in the other countries mentioned above, as well as the USA (18.9% and 11.9:1, respectively) and Bolivia (10.2% and 5.4:1, respectively) (36, 39, 40). The phenomenon of higher OPL/OPO ratio can be explained by either an elevated LA content or a lower OA content. The OA content (32.0%) reported in this study is in accordance with other studies conducted in China (34, 41) and did not significantly differ from those observed in most Western countries (7, 36, 39, 40).

### Associations of Dietary Intake With the Fatty Acid Content in Breast Milk

The two sources of FAs in breast milk—endogenous synthesis in the mammary gland and uptake from maternal plasma—are both subjected to inter-individual biological variations, mainly affected by maternal diet (42, 43). Our study revealed an unbalanced maternal nutrient intake characterized by excessive fat intake for 76.9% of the lactating mothers compared with the dietary reference intakes for lactating women. This excessive fat intake concurs with two studies performed in southeast and northeast China (44, 45) and may be related to the nature of the Chinese postpartum dietary custom “Zuo yuezi.” This postpartum practice refers to increasing the consumption of high-fat and protein-rich foods, such as chicken soup, pig's trotter soup, etc. Opposite to this dietary pattern of Chinese lactating mothers, influenced both by cultural and social contexts, studies from Western countries, such as Spain, UK, USA, and Brazil, showed little change in the dietary pattern from preconception to the postpartum period (22, 23, 46, 47). In our study, excessive consumptions of nuts (55.8%) and cooking oils (40.4%) are likely to be major contributors to the high-fat intake of the lactating mothers. As for the differences in the fat composition of the four dietary patterns derived from this study, both dietary patterns 1 and 4 are likely to feature a high SFA intake as dairy, livestock, and poultry meat are rich in SFAs. Dietary patterns 2 and 3 may be characterized by a high PUFA intake resulting from the high consumption of freshwater food, or nuts and mixed beans.

Among cooking oils, vegetable oils are a major source of fat in the Chinese diet (40.8%), followed by animal foods (27.8%), vegetable foods (20.9%), and animal oil (10.4%) (48). It was previously proposed that a higher consumption of vegetable oils dominantly accounted for the higher proportion of LA in breast milk (40). Soybean oil is highly enriched with LA and is ranked first among the total consumption of Chinese cooking oils (44.0%) (49). Therefore, it is one of the main dietary sources of LA in China. However, no correlation was found with the cooking oils in this study, which may be due to the incomplete record of the vegetable oil categories. Additional sources for LA (up to 56.7%) and broadly consumed Chinese food attributes are soybeans and diverse soybean products (50). Jiang et al. (51) proposed that higher bean consumption might contribute to an increased LA content in breast milk. Our study first directly observed that the consumption of soybeans and soybean products during the preceding month was positively associated with the LA content.

Our study also suggested an inverse association between the LA level and seafood consumption. A possible explanation might well be the competition for the delta-6 desaturase enzyme between LA and ALA. This enzyme is responsible for the conversion of both LA to *n*-6 long-chain (LC)-PUFAs and ALA to *n*-3 LC-PUFAs (52). The competition for the delta-6 desaturase enzyme between LA and ALA can be influenced by dietary intake of the precursors, LA and ALA (53, 54). We infer that a low intake of non-essential *n*-3 FA from seafood, such as eicosapentaenoic acid (EPA) and docosahexaenoic acid (DHA), might enhance the endogenous conversion of ALA to *n*-3 LC-PUFAs, similar to the effect of a high intake of ALA. This enhancement is likely to suppress the conversion of LA to *n*-6 LC-PUFAs due to the competition between LA and ALA. The suppressed conversion of LA results in its accumulation in breast milk. A low *n*-3 FA intake from seafood may partially result from the Chinese custom “Zuo yuezi,” which may also be one of the reasons for the huge discrepancy with the recommended consumptions of seafood and freshwater food observed for the lactating mothers of this study. Nevertheless, more research is warranted in this field.

Evaluation of the associations of the OA content of breast milk with the dietary intake of Chinese lactating mothers indicated a negative association between the OA content and cooking oil consumption. This can be most probably ascribed to the fairly low proportion of high-OA oil intake, such as olive oil. Likewise, the high intake of foods poor in OA, including soybeans and soybean products, livestock and poultry meat, and nuts, would be the reason for the observed inverse associations.

Neither the content of LA nor OA in our breast milk samples was significantly correlated with any of the dietary patterns. One Chinese research performed in Jilin Province found that lactating mothers adhering to a dietary pattern featuring mushrooms and algae, meat, and marine products had a higher proportion of *n*-6 PUFAs in breast milk, despite no further analysis on LA (45). To our understanding, only the above study has analyzed the association of diet patterns with the fat components of breast milk. Noteworthy is one study which suggested that maternal body stocks of FAs had a greater influence on breast milk FA composition than the estimated diet during the puerperium (53). Based on this, it is conceivable that the dietary pattern featuring a preference for vegetable foods including soybeans and soybean products, embedded in China, relates to a unique phenomenon of higher OPL in breast milk during a longer period than that studied.

PA, another abundant fatty acid (19.6%) in human milk, accounted for over half of the total SFAs. The PA content in Chinese human milk was not different from that of Western countries (36, 39, 40). Interestingly, the PA content was found to be positively associated with the dietary pattern featuring high consumption of livestock and poultry meat, which needs to be explored further. It has been shown that the majority of PA in human milk (60–86%) is esterified to the middle position (sn-2) of the glycerol backbone (55, 56), which has important physiological and metabolic implications in breastfed infants. Several studies showed that increasing the content of sn-2-palmitate in infant formulas facilitates FA and calcium absorption, improves the stool consistency and intestinal microbiome, as well as reduces crying (57–60). However, research showing the positive influence of a high-sn-2 palmitate formula contained synthetic sn-2 palmitate with a higher level of OPO than OPL. Although it is reasonable to assume that both will exert the same physiological benefits on the infant, the effect of formula enriched with OPL over OPO remains to be explored. Moreover, our observation of the LA/ALA ratio (16.3:1) exceeding the current standard (5:1–15:1) of Chinese infant formula marketed was established based on the European reference (61). In addition, correlations between the dietary intakes and the FA levels in human milk could be modified by gene polymorphisms (62). Therefore, further investigation is required to determine whether the interaction of genetic factors with the dietary effects on the FA composition of breast milk leads to higher OPL in Chinese breast milk and makes it more suitable for Chinese infants.

Three limitations should be addressed in our study. Firstly, during investigation of the 24-h dietary recall, many participants were unable to clarify the specific kind of cooking vegetable oil or reported using mixed oils. This, to some extent, limited our further analysis on the effect of each vegetable oil on the composition of breast milk fat. In addition, there is still lack of data regarding the FA contents of many foods in the Chinese Food Composition Table, thus hindering direct evaluation of the associations between the FA composition of breast milk and that of the maternal diet. It would be better to apply methods that can identify cooking oil species, such as chemical analytical dietary survey, to profile the dietary intake of fatty acids in the future. Secondly, in this pilot study recruitment of lactating women was focused on the Beijing area to minimize heterogeneity. As such, future research on a diversified and larger population is required to confirm these results, taking possible interactive and confounding factors into consideration. Thirdly, a one-time 24-h dietary recall could not picture the variations and long-term nutrient intakes of a general population. The one-time 24-h recall was used in this study because previous studies have emphasized the relevance and importance of the maternal dietary intake (mainly fat) during the preceding 24 h on human milk fat composition (63). In addition, the diets of lactating mothers during this confinement period are usually considered to have less day-to-day variations on a short-term basis. Also, a 21-item semi-FFQ was used to assess the diets of the lactating women 1 month prior to the human milk collection, which could reflect the medium-/long-term food intakes and dietary habits. Seven-day food records could be adopted for a more accurate nutritional assessment in subsequent research.

This study confirms OPL as the predominant TAG species in Chinese breast milk, showing evidently high LA levels compared to breast milk in Western countries. The study demonstrates the associations of FAs with maternal dietary intake. Observations of excessive fat intake occurred for 76.9% of the study participants, along with low protein consumptions, indicating a need to follow a more balanced diet for Chinese lactating women, especially emphasizing less fat intake, which is affected by the unique postpartum dietary custom. More seafood consumption was also needed for Chinese lactating women as a huge discrepancy with the recommendation existed in this study; the ratio of 4–6:1 for *n*-3 LC-PUFA to *n*-6 LC-PUFA intake is recommended. This work is a pilot study, and additional research is needed to reveal the origins of the heterogeneity in breast milk fat composition and to explore the metabolic fate and functional significance of OPL, the main TAG consumed by Chinese babies.

## Data Availability Statement

The raw data supporting the conclusions of this article will be provided by the authors upon request.

## Ethics Statement

The studies involving human participants were reviewed and approved by Medical Ethics Research Board of Peking University. The patients/participants provided their written informed consent to participate in this study.

## Author Contributions

AB, GB-D, YZ, and AZ conceived the research and designed the study. WW and AZ carried out the recruitment of participating mothers and were responsible for breast milk and data collection. VV and YO carried out the human milk lipid analysis. WW, AB, and AZ were responsible for data analysis and interpretation of the results, and wrote the manuscript with inputs from all authors.

## Conflict of Interest

AB, VV, and YO are employed by the company IFF. GB-D was employed by the company Enzymotec at the time during which the research work was undertaken. The remaining authors declare that the research was conducted in the absence of any commercial or financial relationships that could be construed as a potential conflict of interest.

## Abbreviations

TAG: triacylglycerol
FA: fatty acid
OPO: oleic–palmitic–oleic
OPL: oleic–palmitic–linoleic
METs: metabolic equivalents
FFQ: food frequency questionnaire
HPLC: high-performance liquid chromatography
UPLC: ultra-performance liquid chromatography
ACN: acyl carbon number
DB: number of double bonds
PA: palmitic acid
OA: oleic acid
LA: linoleic acid
ALA: alpha-linolenic acid
PUFA: polyunsaturated fatty acid.
