# Supplementary material for: Chinese Breast Milk Fat Composition and Its Associated Dietary Factors: A Pilot Study on Lactating Mothers in Beijing
Source: Front Nutr. 2021 May 28;8:606950. doi: 10.3389/fnut.2021.606950 (PMC8193926; doi:10.3389/fnut.2021.606950)
Supplement: Supplementary file 1 [file Data_Sheet_1.docx]

Chinese Breast Milk Fat Composition and its Associated Dietary Factors: a Pilot Study on Lactating Mothers in Beijing

Supplementary Material

# Supplementary Figures and Tables

## Supplementary Figures


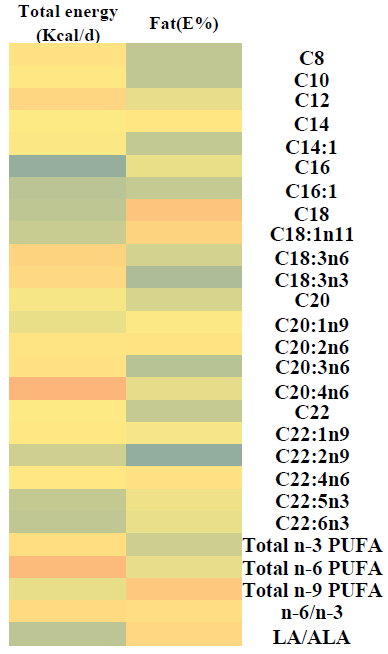

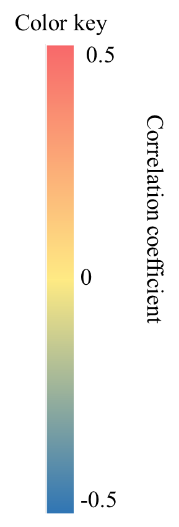


**Supplementary Figure 1(A).** Heat map of correlation coefficient between intake of total energy, fat during the preceding 24 hours and fatty acid contents in breast milk. Legend: The rows present fatty acid in breast milk. Column represents intake of total energy, fat during the preceding 24 hours. *denotes p value＜0.05.


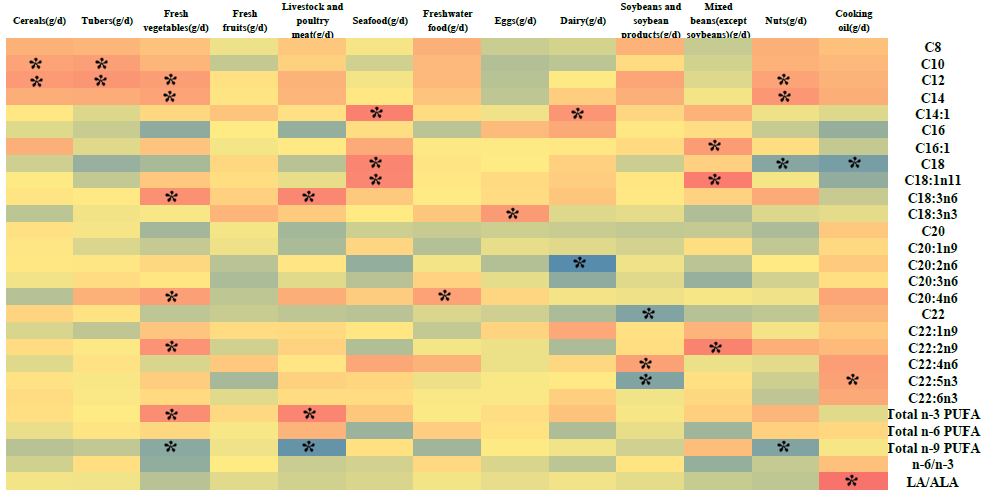

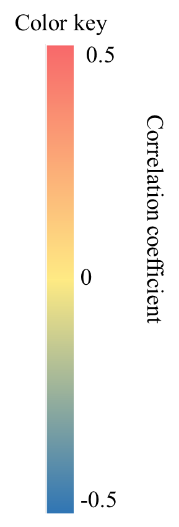


**Supplementary Figure 1(B).** Heat map of correlation coefficient between consumption of food groups during the preceding month and fatty acid content in breast milk. Legend: The rows present fatty acid in breast milk. Column represents food groups consumed during the preceding month. *denotes p value＜0.05.


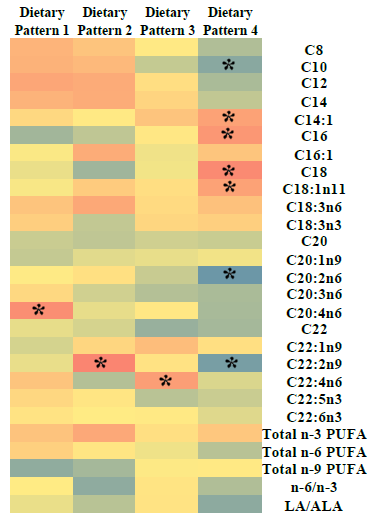

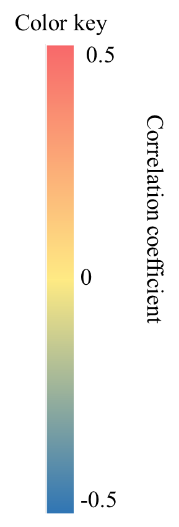


**Supplementary Figure 1(C).** Heat map of correlation coefficient between dietary patterns during the preceding month and fatty acid contents in breast milk. Legend: The rows present fatty acid in breast milk. Column represents dietary patterns during the preceding month. *denotes p value＜0.05.

## Supplementary Tables

**Supplementary Table 1.** Factor loading in dietary pattern derived from food groups consumed over the preceding month

| Food groups | Dietary Pattern 1 | Dietary Pattern 2 | Dietary Pattern 3 | Dietary Pattern 4 |
| --- | --- | --- | --- | --- |
| Cereals | -0.081 | 0.053 | 0.476 | 0.079 |
| Tubers | -0.246 | 0.742 | -0.006 | -0.110 |
| Fresh vegetables | 0.145 | 0.589 | 0.568 | 0.339 |
| Fresh fruits | 0.718 | 0.080 | 0.181 | 0.060 |
| Livestock and poultry meat | 0.339 | 0.368 | 0.269 | 0.738 |
| Seafood | 0.521 | 0.477 | -0.133 | 0.247 |
| Freshwater food | 0.062 | 0.841 | 0.088 | 0.040 |
| Eggs | 0.358 | -0.045 | 0.071 | 0.014 |
| Dairy | 0.605 | -0.030 | -0.224 | -0.055 |
| Soybeans and soybean products | 0.559 | -0.043 | 0.322 | -0.083 |
| Mixed beans(except soybeans) | 0.086 | -0.088 | 0.643 | -0.222 |
| Nuts | 0.198 | 0.108 | 0.734 | 0.159 |
| Cooking oil | -0.334 | -0.062 | 0.146 | 0.457 |
